# Hematological Changes in Women and Infants Exposed to an AZT-Containing Regimen for Prevention of Mother-to-Child-Transmission of HIV in Tanzania

**DOI:** 10.1371/journal.pone.0055633

**Published:** 2013-02-06

**Authors:** Judith Ziske, Andrea Kunz, Julius Sewangi, Inga Lau, Festo Dugange, Andrea Hauser, Wolf Kirschner, Gundel Harms, Stefanie Theuring

**Affiliations:** 1 Institute of Tropical Medicine and International Health, Charité-Universitätsmedizin Berlin, Germany; 2 Regional AIDS Control Program Mbeya Region, Ministry of Health and Social Welfare, Mbeya, Tanzania; 3 Kyela District Hospital, Ministry of Health and Social Welfare, Kyela, Tanzania; 4 Forschung Beratung+Evaluation, Berlin, Germany; University of Cape Town, South Africa

## Abstract

**Introduction:**

Tanzanian guidelines for prevention of mother-to-child-transmission of HIV (PMTCT) recommend an antiretroviral combination regimen involving zidovudine (AZT) during pregnancy, single-dosed nevirapine at labor onset, AZT plus Lamivudine (3TC) during delivery, and AZT/3TC for 1–4 weeks postpartum. As drug toxicities are a relevant concern, we assessed hematological alterations in AZT-exposed women and their infants.

**Methods and Materials:**

A cohort of HIV-positive women, either with AZT intake (n = 82, group 1) or without AZT intake (n = 62, group 2) for PMTCT during pregnancy, was established at Kyela District Hospital, Tanzania. The cohort also included the infants of group 1 with an *in-utero* AZT exposure ≥4 weeks, receiving AZT for 1 week postpartum (n = 41), and infants of group 2 without *in-utero* AZT exposure, receiving a prolonged 4-week AZT tail (n = 58). Complete blood counts were evaluated during pregnancy, birth, weeks 4–6 and 12.

**Results:**

For women of group 1 with antenatal AZT intake, we found a statistically significant decrease in hemoglobin level, red blood cells, white blood cells, granulocytes, as well as an increase in red cell distribution width and platelet count. At delivery, the median red blood cell count was significantly lower and the median platelet count was significantly higher in women of group 1 compared to group 2. At birth, infants from group 1 showed a lower median hemoglobin level and granulocyte count and a higher frequency of anemia and granulocytopenia. At 4–6 weeks postpartum, the mean neutrophil granulocyte count was significantly lower and neutropenia was significantly more frequent in infants of group 2.

**Conclusions:**

AZT exposure during pregnancy as well as after birth resulted in significant hematological alterations for women and their newborns, although these changes were mostly mild and transient in nature. Research involving larger cohorts is needed to further analyze the impact of AZT-containing regimens on maternal and infant health.

## Introduction

Mother-to-child transmission of HIV has become a relatively rare event in most resource-rich countries, where vertical transmission nowadays occurs in less than 2% of cases [1]. This decline is based on a combination of several strategies, including early maternal diagnosis through routine counseling and HIV testing during antenatal care (ANC), provision of antiretroviral therapy (ART) or of antiretroviral (ARV) prophylaxis, elective Caesarean section and the complete avoidance of breastfeeding. The high requirements for this complex range of measures, such as access for women to a health care system, broad coverage of HIV testing among pregnant women, CD4 cell count monitoring, or affordable and sustainable replacement feeding [2], make it difficult to successfully reduce mother to child transmission of HIV (PMTCT) in resource-limited countries. Indeed, in 2010, ARV coverage for PMTCT was only about 50% in sub-Saharan Africa [3]. The implementation of a single-dose (sd) administration of the non-nucleoside reverse transcriptase inhibitor nevirapine (NVP) to mothers and babies in resource-poor countries has been a considerable step forward in PMTCT. However, although representing a simple, feasible and cost-effective regimen [4], a major problem of sdNVP is the high risk of inducing drug-resistant HIV variants. It has been shown that the addition of nucleoside reverse transcriptase inhibitors, such as Zidovudine (AZT) and Lamivudine (3TC), can significantly reduce this risk [5]. Furthermore, combining several drugs is more effective in reducing HIV-transmission and can result in transmission rates as low as 6.5% at six weeks postpartum [6].

Since 2006, the World Health Organization (WHO) PMTCT guidelines for resource-poor settings follow those findings and recommend a sequential combination prophylaxis, including antenatal AZT intake, sdNVP during labor and intra/postpartum AZT/3TC. Despite the clear advantages of this regimen in terms of efficacy and reduction in NVP resistance, it has nevertheless several drawbacks. As drug intake is supposed to last from pregnancy until the postpartum period, requiring different drugs at specific points in time, this prolonged and complex process can make adherence difficult [2], [7]. Another important issue is that a combination prophylactic regimen obviously results into a much higher drug burden for mothers and infants than the previously recommended regimen. Previous retro- and prospective studies have shown that AZT interferes with hematopoiesis, resulting in decreased levels for several cell lineages in pregnant women [8], [9]. Other studies have shown an impact on hematopoiesis with varying persistence in infants exposed to AZT *in utero* or postnatally [10], [11], [12], [13]. Connor *et al.* [12] and Sperling *et al.* [14] found a transiently lower hemoglobin level that resolved within the first six to 12 weeks of age. Regarding other cell lineages, especially granulocytes, Le Chenadec *et al.* found that decreased levels persisted up to the age of 18 months [11]. As granulocytes are crucial for the immune response, with deficiency often leading to severe bacterial infections, and anemia can have life-threatening potential, monitoring is a crucial aspect, particularly in settings where treatment options are limited.

The United Republic of Tanzania, one of the poorest countries in the world [15], is also one of the countries most affected by the global HIV/AIDS epidemic. The general HIV prevalence is estimated to be 6%, while the prevalence of HIV in pregnant women is estimated to be 10% in major urban areas and 6% in less densely populated regions [16]. In 2008, Tanzania changed its PMTCT standard recommendation from sdNVP to a combination regimen in accordance with the 2006 WHO guidelines.

The aim of this study was to assess the potential hematological toxicity of the combination PMTCT regimen in women and infants in a peripheral setting in Tanzania.

## Methods

### Ethics Statement

The study was approved by the Tanzanian National Institute of Medical Research, by the Mbeya Region Ethical Committee, and by the Ethical Commission of Charité-Universitätsmedizin Berlin. Written informed consent was obtained from all participants, and all data remained confidential.

### Setting, Procedures and Recruitment

An observational prospective follow-up study was conducted from September 2008 until September 2009 at the Kyela District Hospital (KDH) in Mbeya Region, Tanzania to accompany the introduction of ARV combination prophylaxis for MTCT with regard to feasibility and adherence [7]. Within the larger frame of this research, we performed a sub-study assessing hematological alterations linked to this regimen in mothers and infants.

Mbeya is among the regions in Tanzania with the highest HIV prevalence, estimated at around 9% of the general population [17]. KDH is a rural health facility in the south of Mbeya Region. Each month, approximately 130 pregnant women attend the ANC services of KDH for the first time. This hospital has been providing PMTCT services supported by the German Agency for International Cooperation since 2001. Combination ARV prophylaxis was introduced in KDH in March 2008 following the updated 2008 Tanzanian Guidelines.

Routine PMTCT procedures at KDH include voluntary HIV counseling and testing for all new antenatal care (ANC) clients visiting KDH. CD4 cell counts were performed for pregnant women identified as HIV-positive to assess their eligibility for either ART or ARV prophylaxis. Women with CD4 cell counts above 200 cells/mm^3^ (and therefore not requiring ART for their own health) were eligible for ARV prophylaxis. Administration of AZT was initiated at week 28 of gestation or anytime thereafter. During delivery, the women received sdNVP, AZT and 3TC and were given a take-home postpartum tail of AZT for seven days. Women first identified as HIV-positive at the time of delivery, who therefore had no previous ARV intake, were also offered intra/postpartum ARV prophylaxis. Infants received sdNVP within 72 hours after birth and AZT syrup for one week if the mother had taken AZT for at least four weeks during pregnancy and for four weeks if the mother had taken AZT for less than four weeks.

The cohort included HIV-positive pregnant ART-naïve women aged 18 years or older who attended the ANC clinic and/or delivered in the maternity ward at KDH during the study period, who were eligible for ARV prophylaxis according to the guidelines and who had given informed consent. Women enrolled in ANC with pre-delivery AZT intake were assigned to group 1, and those who had no ARV prophylaxis during pregnancy but received drugs during delivery at the KDH maternity ward were assigned to group 2.

Group 1-women were included for the analysis of hematological changes during pregnancy. Infants of women participating in the study were eligible for analysis if they had received prophylaxis according to the guidelines, i.e. group 1-infants with postnatal AZT intake of one week and group 2-infants with four weeks (see graph 1). In accordance with the WHO guidelines for postnatal AZT prophylaxis in infants, the analysis of adverse effects in group 1-infants only included those with at least four weeks *in utero* exposure.

### Samples and Data Collection

Data on the socio-demographic and clinical background of the women were collected through standardized, structured questionnaires for the stages of ANC, delivery and postpartum follow-up. The questionnaires were developed and pretested by the authors. Self-observed adverse events, adherence to ARV prophylaxis, and concomitant drug use were recorded at regular intervals throughout pregnancy (group 1), delivery (group 1 and 2) and during the postpartum period (groups 1 and 2). Maternal blood samples from group 1 were taken weekly in the first month of AZT intake, monthly throughout pregnancy, once at delivery, and at months one and three post-delivery. Blood was drawn from group 2 participants at delivery and again monthly during the first three months postpartum. Laboratory analysis included full blood counts for all samples, and CD4 cell counts were conducted with samples taken during the first ANC visit (group 1) and at delivery (groups 1 and 2). The comparability of the groups was confirmed with regard to the socio-demographic situation, CD4 cell count as well as malarial symptoms and malarial prophylaxis.

For the infants, cord blood was taken at delivery and further blood samples were taken during follow-up visits at one and three months of age. Full blood cell counts were performed for both mothers and infants. HIV-PCR was performed with samples from newborns taken at delivery and at one month of age and HIV-infected infants were excluded from the analysis as HIV infection itself causes hematological changes.

Sample taking and data collection in questionnaires and forms was performed by staff within the respective wards at KDH; a trained study nurse supervised this process.

### Statistical Analysis

Data was analyzed using the statistical software Stata version 11. Descriptive analysis of maternal baseline information was performed to characterize the study population. Baseline characteristics of both groups were compared using the student’s t-test, Mann-Whitney-U-test or Fisher’s exact test.

Fixed effects models were used to follow the development of maternal hematological values during AZT intake, taking into account correlation within the woman herself and the variability between women. Coefficients are expressed as an alteration of parameter per day if the linear model was significant. Student’s t-test for independent variables was used to compare hematological values of women by group at birth and to compare hematological parameters of infants at birth and one month of age. For data at three months of age, Mann-Whitney-U-test was performed, taking the dropout rate into account. To classify ARV induced toxicities, tables from the Division of AIDS (DAIDS) were used for grading the severity of adverse events in adults and infants [18]. At birth, hemoglobin levels between 10 g/dl and 8.5 g/dl were defined as mild anemia (grade 1); 8.4 g/dl to 7.5 g/dl as moderate anemia (grade 2); 7.4 g/dl to 6.5 g/dl as severe anemia (grade 3) and hemoglobin levels less than 7.4 g/dl as potentially life threatening anemia (grade 4). Granulocyte counts between 5/nl and 4/nl were defined as grade 1 toxicity; less than 4/nl to 3/nl as grade 2 toxicity; less than 3/nl to 1.5/nl as grade 3 toxicity and counts less than 1.5/nl as grade 4 toxicity. For follow-up visits, toxicity thresholds for granulocyte counts and hemoglobin levels were adjusted to age according to the DAIDS tables. Frequencies of adverse events were compared using Fisher’s exact test. Prematurity was defined as delivery before 37 weeks of gestation. The Kaplan-Meier approach was used to estimate the cumulative proportion of infected infants at six weeks of age, representing short term efficacy [19]. As recommended by the Ghent Group [19], [20], any PCR result collected from infants between 29 and 60 days of age in combination with earlier results were used for this estimation. A p-value below 0.05 was considered statistically significant.

## Results

### Study Population

**Figure 1:**
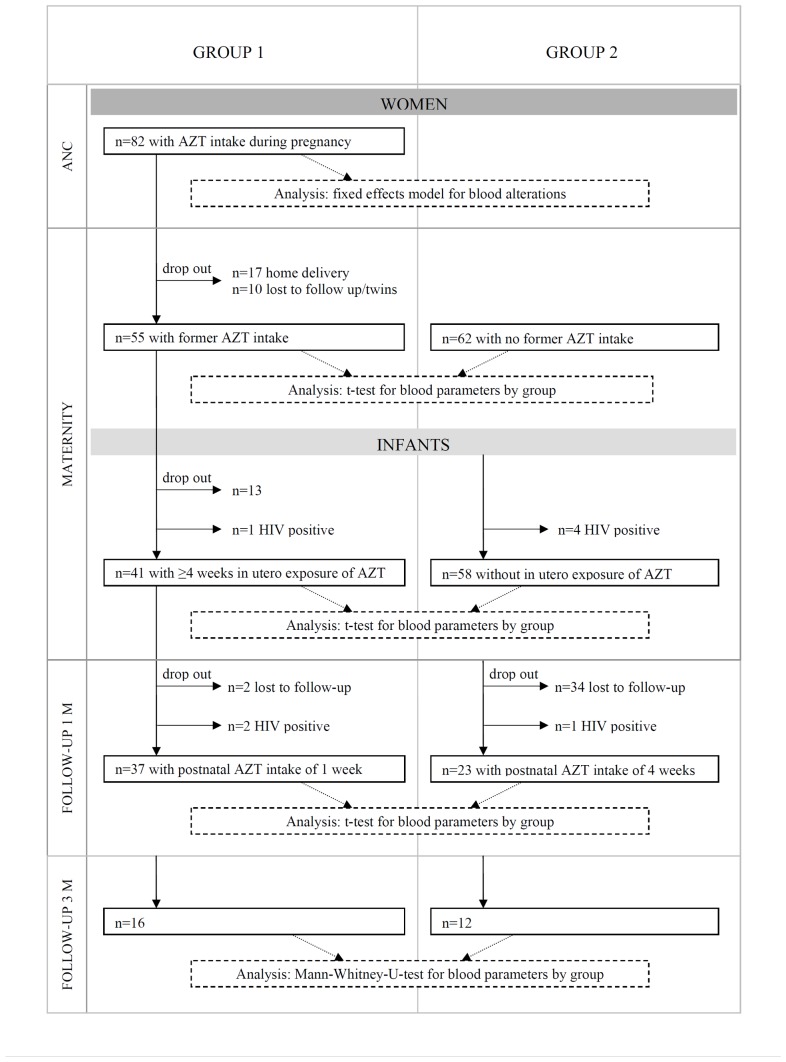
Flow chart of study cohort. Study population and applied statistical tests during antenatal care visits, delivery and follow-up visits at one month and three months post-delivery.

During the study period, 1395 pregnant women were counseled and tested for HIV infection at the ANC clinic of KDH [7]. HIV infection was diagnosed in 220 women (15.8% of all tested), of whom 121 met the eligibility criteria for this study. The study population during all stages of observation is explained in Figure 1.

Eighty-two women had a pre-delivery AZT intake of at least one week and were therefore assigned to group 1. The median CD4 cell count at enrolment in this group was 390 (inter-quartile range [IQR]: 267 to 515) cells/mm^3^ and the median gestational age at start of AZT intake was 29.1 (IQR: 28.0 to 32.2) weeks. Hematological parameters and the specifications of adverse events of these 82 women were analyzed during pregnancy. Fifty-five women of group 1 delivered at KDH with 41 infants born within this group having been exposed to AZT *in utero* for at least four weeks and identified as HIV-negative at birth. The median duration of AZT exposure was 57 (IQR: 43 to 71) days during pregnancy and the median CD4 cell count in mothers at delivery in this group was 350 (IQR: 252 to 490) cells/mm^3^. Thirty-nine of the 41 mother-infant pairs returned for follow-up after one month. Two of the 39 infants were tested HIV positive at that point and were excluded from the analysis. At one month of age, 37 infants remained for assessing hematological alterations. Sixteen group 1-infants were available for blood analysis upon their three month return visit.

In the same period, 62 women meeting the inclusion criteria were enrolled at the time of delivery and assigned to group 2. The median CD4 cell count in these women was 262 (IQR: 194 to 474) cells/mm^3^. Four infants of this group were identified as HIV-positive at delivery and were excluded from the cohort, leaving 58 infants available for the analysis at the time of birth. Twenty-three HIV-negative infants remained for analysis at one month of age, and 12 were returned for follow-up at three months.

Baseline characteristics of both groups, including age, weight, years of education, marital status and malarial symptoms did not differ significantly among the two groups (tables 1 and 2).

**Table 1:** Baseline characteristics of mothers of group 1 and 2.

	Group 1	Group 2	p
	Enrolment in antenatal clinic/pre-delivery AZT intake	Enrolment in maternity ward/no pre-delivery AZT intake	
**Total number of pregnant women included**	82	62	
**Pregnancy week at start of AZT intake [median (IQR)]**	29.1 (28.0–32.2)		
**CD4 at enrolment in antenatal clinic [median (IQR) cells/µl)]**	390 (267–515)		
**Place of delivery [no. (%)]:**			
Maternity ward	55 (67%)	62 (100%)	
Home delivery	17 (21%)^a^	0	
Lost to follow-up	10 (12%)^a^	0	
**Marital status: married [%]:**	74.5%	76.7%	0.96^b^
**Years of education [median (IQR) years]**	7 (7–7)	7 (7–7)	0.20^b^
**Travel minutes to hospital [median (IQR)]**	30 (30–60)	30 (30–60)	0.35
**Household number [median (IQR)]**	4 (3–5)	3 (2–5)	0.51
**Number of children [median (IQR)]**	2 (1–3)	1 (0.5–2)	0.40
**Age at enrolment [median (IQR) years]**	28 (24–30)	25 (23–29)	0.58
**Weight at enrolment [median (IQR) kg]**	60 (54–65)	57 (53–65)	0.64
**Height [median (IQR) cm]**	158 (154–160)	157 (152–160)	0.93
**Gravida [median (IQR)]**	3 (2–3)	3 (2–3)	0.61
**Para [median (IQR)]**	2 (1–2)	2 (1–3)	0.98

a Excluded from below socio-demographic comparison.

b Mann-Whitney-U-test; all other compared by t-test.

**Table 2:** Baseline characteristics of infants of group 1 and 2.

	Group 1	Group 2	p
	Enrolment in antenatal clinic/pre-delivery AZT intake	Enrolment in maternity ward/no pre-delivery AZT intake	
**No AZT intake during pregnancy [no]**		62	
**At least four weeks antenatal AZT/no twins/available blood** **count results [no]**	42		
**HIV positive children at delivery [% (no)]**	2.4% (1/41)	8.5% (4/47)	
**Children enrolled in study [no]**	41	58	
**CD4 of mothers at delivery [median (IQR) cells/µl; no.]**	350 (252–490); 25	262 (194–474); 40	0.26
**Duration of prenatal AZT exposure [median (IQR) days]**	57 (43–71)	0	
**Duration of AZT syrup intake of infants [days]**	7	28	
**Mode of delivery: section [%]**	7.5	11.1	0.41^b^
**Preterm infants [%]**	9.8	3.5	0.20^b^
**Sex: female [%]**	46.3	54.4	0.28^b^
**Weight [median (IQR) g]**	3100 (2840–3450)	3200 (2900–3500)	0.67
**Apgar newborns at 1 minute**	9 (8–9)	9 (8–9)	0.63^a^
**Apgar newborns at 5 minutes**	10 (10–10)	10 (10–10)	0.28^a^
**Frequency of symptom maternal fever at delivery [%]**	2.4	0	0.42
**Median no. of sulphadoxine-pyrimeth. doses** **during pregnancy**	2 (2–2)	2(1–2)	0.10^a^

a Mann-Whitney-U-test.

b Fisher’s exact test; all other compared by t-test.

### Hematological Alterations in Women

Eighty-two women in group 1 had an AZT intake of at least one week and were included in the analyses of blood alterations. Coefficients for alterations in blood components over time were estimated with a fixed effects model for the duration of AZT intake. Mean corpuscular volume (MCV) (coef.: 0.0636fl per day, p<0.001), red distribution width (RDW) (coef.: 0.0655% per day, p<0.001) and platelet count (coef.: 1.0309/nl per day, p<0.001) increased significantly over the time of AZT intake. Red blood count (RBC) (coef.: −0.0040*10^6^/µl per day, p<0.001), white blood cell count (WBC) (coef.: −0.0119/nl per day, p<0.001) and granulocyte count (coef.: −0.0123/nl per day, p<0.001) decreased significantly over time. A significant decrease with a subsequent increase after day 32 of AZT intake was shown for hemoglobin values (p<0.05). Comparing group 1 and 2 at the point of delivery, differences between median hemoglobin and median granulocyte count were not statistically significant. Median red blood cell count was significantly lower in women of group 1 (3.66/µl vs. 4.04/µl, p<0.05), whereas MCV (87fl vs. 79fl, p<0.001) and median platelet count (367000/mm^3^ vs. 273500/mm^3^, p<0.05) were significantly higher in group 1-women with antenatal AZT intake. No significant differences in hematological parameters were observed at one month and three months post-delivery. Detailed results are shown in the supplementary Figure S1.

### Hematological Alterations in Infants

At birth, infants of group 1 with prenatal AZT exposure showed significantly lower median hemoglobin (13.3 g/dl vs. 15.2 g/dl, p<0.001) and median RBC (3.7/µl vs. 4.5/µl, p<0.001) levels compared to those of group 2. The median MCV (106fl vs. 100fl, p<0.001) and RDW (17.1% vs. 16.2%, p<0.01) were significantly higher in infants exposed to AZT during the prenatal period. At birth, the overall frequency of anemia ≥ grade 1 was 26%, with a significantly higher frequency in infants with antenatal AZT exposure (47% group 1 vs. 11% group 2, p<0.001). Two infants with prenatal AZT exposure had severe (grade 3) or potentially life threatening (grade 4) anemia (6% of group 1-infants) at birth compared to zero in group 2.

The median hemoglobin concentrations declined in all infants from 14.3 (IQR: 13.0 to 15.5) g/dl at birth to 11.6 (IQR: 9.9 to 12.7) g/dl at one month of age and to 10.5 (IQR: 9.9 to 11.4) g/dl at three months of age. At one month (IQR: 31–36days) and at three months (IQR: 91–93 days) of age, differences in median hemoglobin and median RBC between both groups were no longer significant.

At one month of age, anemia ≥ grade 1 was observed in 29% of all infants (36% group 1 vs. 20% group 2, p = 0.35), with one of the group 1-infants (3%) showing severe anemia. At three month of age, the overall anemia rate (≥ grade 1) was 58% (46% in group 1 vs. 73% in group 2-infants, p = 0.24).

The median granulocyte count at birth was significantly lower in infants with AZT exposure during pregnancy (5.0/nl in group 1 vs. 7.3/nl in group 2, p<0.05). At birth, granulocytopenia ≥ grade 1 was observed in 37% of all infants with a significantly higher frequency in group 1-infants (52% in group 1 vs. 26% in group 2, p<0.05). Toxicity ≥ grade 3 was observed in 18% of group 1-infants compared to 9% of group 2-infants, p = 0.31.

The overall median granulocyte count was 6.2 (IQR: 3.9–8.6)/nl and decreased to 2.8 (IQR: 1.6–3.4)/nl at one month of age and to 2.7 (IQR: 1.8–3.3)/nl at three months of age. At one month, the median granulocyte count was significantly lower in group 2-infants compared to group 1-infants (3.2/nl in group 1 vs. 1.7/nl in group 2, p = 0.001). At this age, 19% of all infants presented with granulocytopenia ≥ grade 1, with a significantly higher frequency in group 2-infants (9% in group 1 vs. 33% in group 2, p = 0.039). Toxicity ≥ grade 3 was observed in 6% of group 1-infants and 10% of group 2-infants, p = 1.0.

**Figure 2:**
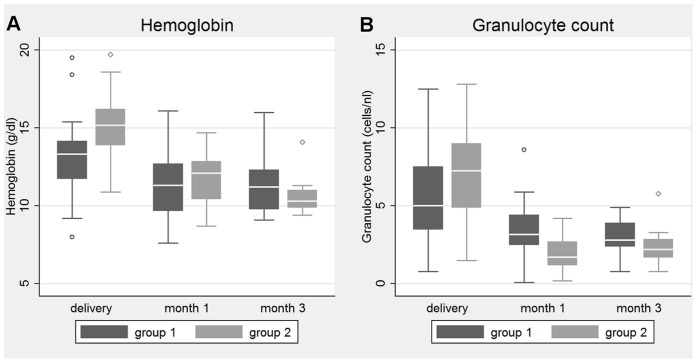
Hemoglobin and granulocyte count in infants at birth, one month and three months of age. A. At birth, group 1-infants with prenatal AZT exposure presented with significantly lower median hemoglobin (13.2 g/dl vs. 15.2 g/dl, p<0.001) than group 2-infants. At one month and at three months of age, differences in median hemoglobin between both groups were no longer significant. B. The median granulocyte count at birth was significantly lower in infants with AZT exposure during pregnancy (5.1/nl in group 1 vs. 7.3/nl in group 2, p<0.05). At one month of age, the median granulocyte count was significantly lower in group 2-infants compared to group 1-infants (3.2/nl in group 1 vs. 1.7/nl in group 2, p = 0.001). No statistically significant differences in granulocyte count were observed at three months of age.

The hematologic parameters of infants in group 1 and group 2 are summarized in table 3 and figure 2. Frequency of anemia and granulocytopenia are shown in table 4.

**Table 3:** Hematological parameters of infants by group at birth, 4–6 weeks of age and 12 weeks of age.

	Birth			Week 4–6 (IQR: 31–36; n = 62)			Week 12 (IQR: 91–93; n = 31)		
	group 1	group 2	p	group 1	group 2	p	group 1	group 2	P^a^
**RBC, median**	3.7 (3.2–4.0)	4.5 (4.2–4.8)	**<0.001**	3.6 (3.0–4.0)	3.7 (3.4–4.1)	0.58	4.4 (4.1–4.5)	4.1 (3.9–4.5)	0.18
**(IQR), 10^6^/uL**			n = 78			n = 51			n = 24
**Hemoglobin,** **median**	13.3 (11.8–14.2)	15.2 (13.9–16.2)	**<0.001**	11.3 (9.7–12.7)	12.1 (10.5–12.9)	0.49	11.2 (9.8–12.3)	10.3 (9.9–11.0)	0.6
**(IQR), g/dl**			n = 78			n = 51			n = 24
**MCV, median**	106 (101–114)	100 (97–105)	**0.001**	95 (89–101)	92 (89–94)	0.27	74 (70–81)	75 (72–78)	0.47
**(IQR), fl**			n = 78			n = 51			n = 24
**RDW, median**	17.1 (16.2–18.1)	16.2 (15.6–16.9)	**0.008**	17.1 (16.8–18.3)	15.7 (14.8–17.1)	**0.002**	15.9 (15.6–16.9)	16.0 (14.6–16.6)	0.54
**(IQR), %**			n = 75			n = 51			n = 24
**WBC, median**	10.4 (8.2–13.6)	12.8 (10.9–17.1)	0.07	9.5 (7.6–11.5)	8.5 (6.3–10.9)	0.35	10.6 (8.8–14.7)	8.9 (6.4–11.4)	0.11
**(IQR),/nL**			n = 79			n = 53			n = 25
**Granulocytes,** **median**	5.0 (3.5–7.5)	7.3 (4.9–9.0)	**0.042**	3.2 (2.5–4.4)	1.7 (1.2–2.7)	**0.001**	2.8 (2.4–3.9)	2.2 (1.7–2.9)	0.18
**(IQR),/nL**			n = 79			n = 53			n = 25
**Lymphocytes,** **median**	4.0 (3.5–6.2)	4.9 (3.2–6.4)	0.42	4.8 (3.9–7.3)	5.3 (4.1–8.3)	0.62	6.8 (5.9–10.2)	6.1 (3.7–7.7)	0.18
**(IQR),/nL**			n = 79			n = 53			n = 25
**Monocytes,** **median**	1.0 (0.6–1.6)	1.3 (0.7–1.9)	0.42	0.9 (0.7–1.3)	0.9 (0.7–1.3)	0.73	0.8 (0.6–1.4)	0.8 (0.5–1.1)	0.16
**(IQR),/nL**			n = 79			n = 53			n = 25
**Platelets,** **median**	375 (303–436)	326 (245–386)	0.09	397 (248–493)	420 (216–533)	0.79	440 (284–568)	403 (215–569)	0.64
**(IQR),/nL**			n = 76			n = 49			n = 23

a Mann-Whitney-U-test, all other compared by t-test.

**Table 4:** Frequency of anemia and granulocytopenia in infants of group 1 and 2.

	Birth (n = 78)			Month 1 (n = 51)			Month 3 (n = 24)		
Anemia	group 1	group 2	P^a^	group 1	group 2	P^a^	group 1	group 2	P^a^
**grade ≥1**	15 (46.9%)	5 (10.9%)	**0.001**	11 (35.5%)	4 (20.0%)	0.35	6 (46.2%)	8 (72.7%)	0.24
**grade ≥3**	2 (06.3%)	0 (00.0%)	0.17	1 (03.2%)	0 (00.0%)	1	0 (00.0%)	0 (00.0%)	1
	**birth (n = 79)**			**month 1 (n = 53)**			**month 3 (n = 25)**		
**Granulocytopenia**	**group 1**	**group 2**	**P** a	**group 1**	**group 2**	**P** a	**group 1**	**group 2**	**P** a
**grade ≥1**	17 (51.5%)	12 (26.1%)	**0.033**	3 (09.4%)	7 (33.3%)	**0.039**	1 (07.7%)	2 (16.7%)	0.59
**grade ≥3**	6 (18.2%)	4 (08.7%)	0.31	2 (06.3%)	2 (09.5%)	1	0 (00.0%)	0 (00.0%)	1

a Fisher’s exact test.

### Transmission Rate

HIV-DNA PCR was performed in 88 infant blood samples at birth and in 63 infant blood samples at six weeks of age.

The *in utero* transmission rate tested at birth was 2.4% (CI: 0.35–16.0) in group 1-infants and 8.5% (CI: 3.3–21.1) in group 2-infants. The transmission rate estimated by Kaplan-Meier at six weeks of age was 7.7% in group 1 (CI: 2.5–22.1) and 12.5% (CI: 5.2–28.5) in group 2-infants.

## Discussion

Focusing on hematological parameters in pregnant women and their infants up to the age of three months in a rural Tanzanian setting, this study was conducted to assess the impact of antiretroviral prophylaxis involving treatment of both mothers and infants with AZT.

Our evaluation was performed within the frame of an observational study in which all patients received antiretroviral prophylaxis as recommended by WHO guidelines. The two subgroups of our cohort were comparable with regard to the clinical and socio-demographic variables tested.

During AZT intake, pregnant women showed a significant decline in granulocyte count and RBC. Hemoglobin decreased within the first four weeks of AZT intake and then increased. Concurrently, a significant increase of MCV, RDW and platelet count was observed. However, at birth, there was no significant difference in hemoglobin between women taking AZT during pregnancy (group 1) and those did not (group 2). The values for MCV, RDW and platelet count were significantly higher in group 1-women at birth and RBC counts were significantly lower.

The transient decrease of hemoglobin is in agreement with previous publications. Briand *et al*. [8] demonstrated a suppression of hemoglobin levels in pregnant women taking AZT. Comparing short versus long exposure during pregnancy, a slight difference in hemoglobin level persisted until the time of delivery whereas differences in the other values resolved by this time.

It is known that platelet counts may generally decrease during pregnancy [21]. However, our fixed effects model showed an increase during AZT treatment of pregnant women, and a significantly higher platelet count in group 1- compared to group 2-women was observed at the time of delivery. This indicates that AZT treatment may increase platelet counts in HIV-infected pregnant women.

AZT has been shown to cross the placenta, reaching a cord-to-maternal blood level ratio of 0.8 [22] and to affect myeloid and erythroid cell lines [23], resulting in a decrease in hemoglobin levels and the number of granulocytes.

Our study found significantly lower hemoglobin levels in infants with intrauterine AZT exposure (group 1) at birth. The effect on hemoglobin was associated with a significantly higher frequency of anemia grade ≥1 at birth. These results agree with those of Connor *et al.* and Sperling *et al.* [12], [14], who both showed significantly lower levels of hemoglobin in infants exposed *in utero* to AZT. In our study, differences in hemoglobin were no longer significant by the age of one month, which is most likely the result of differences in postnatal AZT intake and a faster decrease of hemoglobin in group 2-infants, again agreeing with Connor *et al.* and Sperling *et al*. The overall rate of anemia ≥ grade 1 was 29% with no significant difference between the groups at one month of age. In contrast, Briand *et al.* found significant differences in the frequency of anemia between infants with three days or six weeks of postnatal AZT intake at the age of six weeks. Interestingly, this effect was found to be independent of *in utero* exposure [24].

At birth, infants of group 1 had significantly lower granulocyte counts, accompanied by a significantly higher frequency of granulocytopenia ≥ grade 1. This effect of *in utero* exposure to AZT in infants, including persistence of decreased granulocyte counts up to 18 months after birth, has been described previously [11].

After receiving AZT for one month in the postnatal period, group 2-infants had a significantly lower granulocyte count and significantly higher frequency of granulocytopenia ≥1 than group 1-infants having a postnatal AZT intake of just one week. By the age of one month, 9% of infants had developed a severe granulocytopenia (≥ grade 3), most likely due to *in utero* and postpartal exposure to AZT.

Throughout this study, alterations in the mothers’ and infants’ blood parameters were carefully monitored. However, it must be kept in mind that the routine health care in low-income countries only rarely includes monitoring of hemoglobin and granulocyte counts during AZT-intake. Despite PCR-HIV testing of infantś blood samples at the age of one month being common, economic or logistic constraints preclude the regular monitoring of blood components. It is therefore important to include this lack of monitoring in debates about the potential toxicity of ARVs in the context of peripheral, resource-limited settings.

The transmission rate estimated by Kaplan-Meier was 7.7% in group 1 and 12.5% in group 2-infants at 6 weeks of age. Two randomized, double-blind, placebo-controlled trials have shown that AZT administered in the last four weeks of pregnancy is effective in reducing the transmission rate to 14.7% compared to 24.8% with placebo at six weeks of age [25]. Dabis *et al.* [6] have shown that combining the four-week course of AZT with sdNVP further reduces the six week probability of transmission to 6.5%, a finding largely consistent with our own.

To ensure comparability of the two groups, we checked for differences in disease progression in terms of CD4-cell counts, as well as with regards to socio-demographic aspects such as age, weight, marital status or years of education and found that none of these factors differed significantly between groups 1 and 2. Nevertheless, it cannot be ruled out that there may be other unconsidered factors such as health-seeking behavior that might vary between the two groups and act as confounders.

At the point of birth, group 2-infants, who had no prior AZT exposure during pregnancy, represent a true control group for analyzing the influence on group 1- infants of AZT exposure during pregnancy. However, during follow-up visits at one and three months of age, infants of both groups had different pre- as well as postnatal ARV exposure and are not strictly comparable. It is a limitation of this study that alterations in blood parameters at these time points can therefore not explicitly be assigned to either part of the regimen. However, establishing a control group with no AZT exposure at all would have exceeded the frame of a study that was primarily aimed at describing the hematological effects of PMTCT regimens actually administered in practice.

A further limitation of our study is the high drop-out rate of infants throughout the follow-up period. Loss to follow-up is a common problem for PMTCT services in resource-limited settings [26], [27]. In addition to the parents failing to understand the importance of follow-up visits, especially in the absence of illness, infant death is a major reason for loss to follow-up, as described by Ahoua *et al.* [28]. In our study, no information about the development of infants lost to follow-up exists and cases of undiagnosed virus transmission, side effects or mortality among such infants cannot be ruled out.

In conclusion, this study revealed that AZT exposure during and after pregnancy can cause significant hematologic alterations in women and infants. However, it was also shown that the side effects observed were generally transient and predominantly mild in nature. However, the few cases of severe hematologic toxicity (≥ grade 3) in infants should be taken into serious consideration when planning and implementing antiretroviral PMTCT interventions in structure-limited settings, where surveillance of blood alterations is often not standard practice. Our results demonstrate that monitoring side effects during antiretroviral PMTCT regimens is a crucial factor for intervention safety, and should be a constituent part for any such services. Further research involving larger cohorts and longer follow-up periods are needed to further analyze the impact of regimens involving AZT on maternal and infant health.

## Supporting Information

Figure S1 **Blood values in women of groups 1 and 2 during AZT intake Figures show selected blood values in group 1- and 2-women during AZT intake.** Time points are: initiation of AZT intake (day 0), 7^th^, 14^th^, 21^st^, 28^th^ day of AZT intake, delivery and one month post-delivery. The period between 28^th^ day of AZT intake and delivery differs between women, depending on the gestational stage at initiation of AZT. Group 1-women had antenatal AZT intake, group 2-women were included at delivery and had no antenatal AZT intake. Sample sizes were: n≥70 at beginning, n≥70 at 7^th^day, n≥56 at 14^th^day, n≥62 at 21^st^day, n≥41 at 28^th^ day of AZT intake. At delivery, sample sizes were n≥30 (group 1-women) and n≥54 (group 2 women); at one month post-delivery, sample sizes were n≥39 (group 1-women) and n≥26 (group 2-women). A. A decrease and a subsequent increase in hemoglobin values was shown in group 1- women. No significant difference between the median hemoglobin levels of group 1- and 2-women was observed at birth and one month post-delivery. B. Mean corpuscular volume increased with AZT intake and resulted in statistically significant higher values at delivery (87fl vs. 79fl, p<0.001) in group 1-women. C. Red blood count decreased in the first weeks of AZT intake. Median red blood count was significantly lower in group 1-women (3.66/µl vs. 4.04/µl, p<0.05) at delivery. There was no statistically significant difference at 1 month post-delivery. D. Platelet count increased during the time of AZT intake. At delivery, the median platelet count was significantly higher in women with antenatal AZT intake (367.000/mm^3^ vs. 273.500/mm^3^, p<0.05), although by one month post-delivery the difference was no longer significant. E and F. White blood counts and granulocyte counts decreased during AZT intake. Comparing group 1 and 2 at delivery and one month post-delivery, differences in the median white blood count and median granulocyte count were not statistically significant.
(TIF)
