# Supplementary figures and images for: Hematological Changes in Women and Infants Exposed to an AZT-Containing Regimen for Prevention of Mother-to-Child-Transmission of HIV in Tanzania
Source: PLoS One. 2013 Feb 6;8(2):e55633. doi: 10.1371/journal.pone.0055633 (PMC3566062; doi:10.1371/journal.pone.0055633)

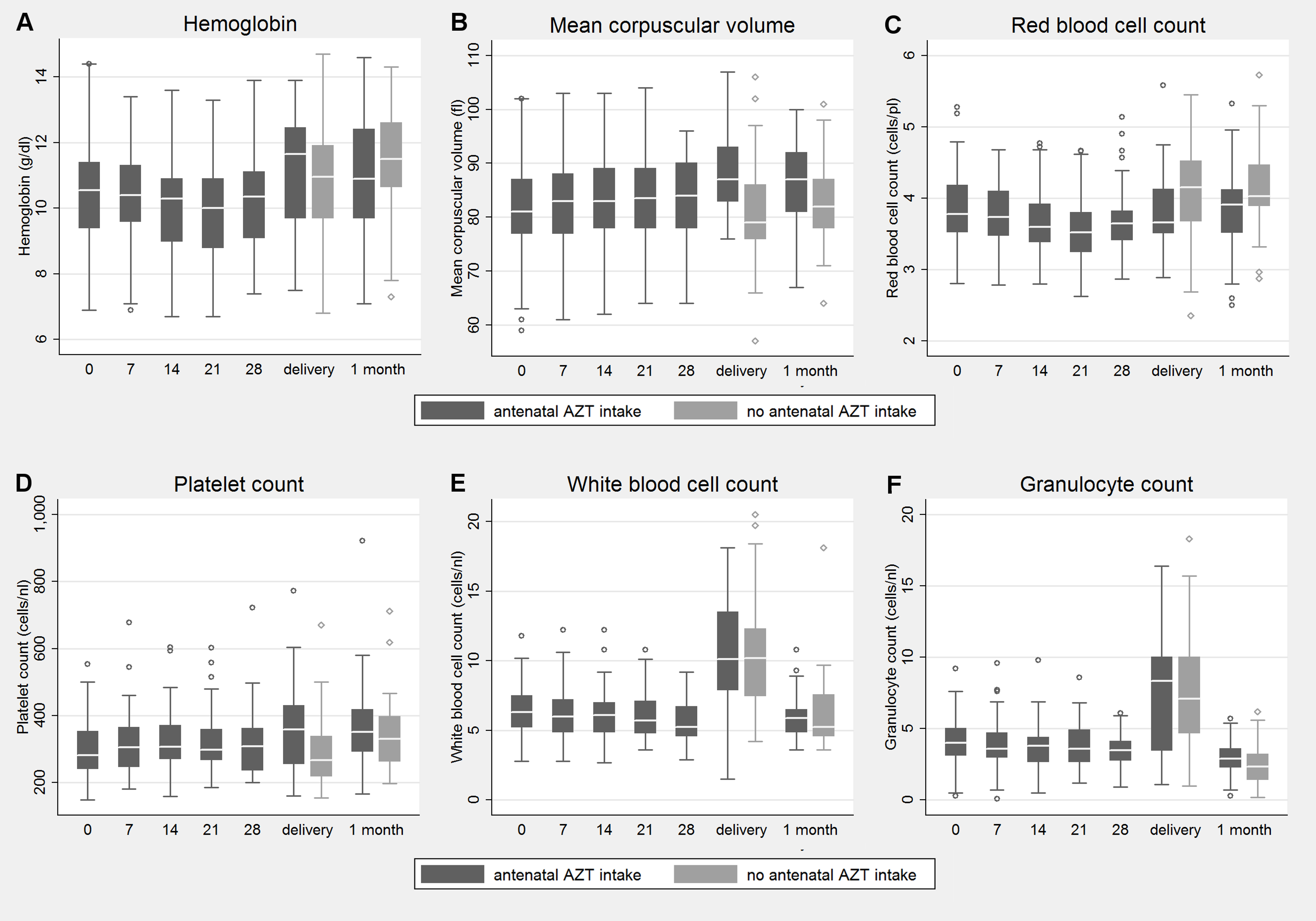

Supplement: Figure S1 — Blood values in women of groups 1 and 2 during AZT intake Figures show selected blood values in group 1- and 2-women during AZT intake. Time points are: initiation of AZT intake (day 0), 7th, 14th, 21st, 28th day of AZT intake, delivery and one month post-delivery. The period between 28th day of AZT intake and delivery differs between women, depending on the gestational stage at initiation of AZT. Group 1-women had antenatal AZT intake, group 2-women were included at delivery and had no antenatal AZT intake. Sample sizes were: n≥70 at beginning, n≥70 at 7thday, n≥56 at 14thday, n≥62 at 21stday, n≥41 at 28th day of AZT intake. At delivery, sample sizes were n≥30 (group 1-women) and n≥54 (group 2 women); at one month post-delivery, sample sizes were n≥39 (group 1-women) and n≥26 (group 2-women). A. A decrease and a subsequent increase in hemoglobin values was shown in group 1- women. No significant difference between the median hemoglobin levels of group 1- and 2-women was observed at birth and one month post-delivery. B. Mean corpuscular volume increased with AZT intake and resulted in statistically significant higher values at delivery (87fl vs. 79fl, p<0.001) in group 1-women. C. Red blood count decreased in the first weeks of AZT intake. Median red blood count was significantly lower in group 1-women (3.66/µl vs. 4.04/µl, p<0.05) at delivery. There was no statistically significant difference at 1 month post-delivery. D. Platelet count increased during the time of AZT intake. At delivery, the median platelet count was significantly higher in women with antenatal AZT intake (367.000/mm3 vs. 273.500/mm3, p<0.05), although by one month post-delivery the difference was no longer significant. E and F. White blood counts and granulocyte counts decreased during AZT intake. Comparing group 1 and 2 at delivery and one month post-delivery, differences in the median white blood count and median granulocyte count were not statistically significant. (TIF) [file pone.0055633.s001.tif]
